# Translation and validation of a Chinese version of the body talk scale for women and men

**DOI:** 10.1186/s40337-023-00884-y

**Published:** 2023-09-11

**Authors:** Jinbo He, Yining Lu, Wesley R. Barnhart, Chanyuan Tang, Hengyue Zhang, Yiqing Zhao, Linda Lin

**Affiliations:** 1 School of Humanities and Social Science, The Chinese University of Hong Kong, Shenzhen, 518172 Guangdong China; 2 Department of Psychology, Bowling Green State University, Bowling Green, OH USA; 3 Department of Psychology, University of Macau, Macau, China; 4 Emmanuel College, 400 The Fenway, Boston, MA USA

**Keywords:** Body talk, Fat talk, Muscle talk, Positive body talk, Validation, Chinese

## Abstract

Body talk has received increasing research attention in recent years, with accumulating evidence supporting the link between body talk and eating and body image disturbances. However, research on body talk in China is still relatively scarce and generally focused on fat talk, especially in women, and much remains unknown about muscle talk and positive body talk for both Chinese women and men. To promote a better understanding of body talk in the Chinese context, the present study adapted the Body Talk Scale (BTS) into Chinese Mandarin (i.e., C-BTS) and evaluated the factor structure and psychometric properties of the C-BTS in Chinese adult women and men. The English version of the BTS was translated into Chinese Mandarin with standard procedures. With 300 Chinese women (*M*_age_ = 29.48 years, *SD* = 7.26) and 300 men (*M*_age_ = 29.36 years, *SD* = 6.81), we examined the factor structure and gender invariance of the C-BTS, as well as internal consistency reliability, test–retest reliability, and construct validity, including convergent, concurrent, and incremental validity of the C-BTS. The results indicated that, consistent with the development study of the BTS, the C-BTS had three subscales (i.e., Negative Fat Talk, Negative Muscle Talk, and Positive Body Talk) and good reliability and validity. The findings demonstrate that the C-BTS can be a useful measure of body talk in both Chinese women and men.

**Supplementary Information:**

The online version contains supplementary material available at 10.1186/s40337-023-00884-y.

## Abstract (plain language summary)

The Body Talk Scale (BTS) measures three types of body talk, including negative fat talk, negative muscle talk, and positive body talk. The present study adapted the English version of the BTS into Chinese Mandarin and examined its psychometric properties in Chinese adult women and men. Results showed that the BTS had adequate reliability and validity in Chinese adults and could be used to assess body talk in Chinese women and men.

**Supplementary Information:**

The online version contains supplementary material available at 10.1186/s40337-023-00884-y.

## Introduction

In China, body dissatisfaction and disordered eating are highly prevalent [1–6], and the prevalence of eating disorders (EDs) continues to increase in recent decades [7]. Given the adverse health consequences (e.g., poor psychological and physical health [8, 9]), body dissatisfaction and disordered eating/EDs are public health concerns in China. Continued efforts are warranted to explore the etiology of body dissatisfaction and disordered eating/EDs in China. One important risk factor contributing to body dissatisfaction, disordered eating, and EDs is body talk [10, 11], which refers to conversations in which people disclose thoughts, emotions, or attitudes about their bodies [12]. Based on the directions (negative vs. positive) and body features included in this discourse (body fat vs. muscle), there are different types of body talk, specifically negative body talk (i.e., talk about one’s body in a disparaging way) and positive body talk (talk one's body in an accepting and appreciative way [13]). Furthermore, negative body talk can be distinguished as fat talk or muscle talk, with fat talk focused on conversations related to body shape/size [14] and muscle talk focused on conversations related to muscularity [15].

### Body talk, body image, and disordered eating

Cumulative evidence suggests that engagement in different types of body talk is differently associated with body image and disordered eating outcomes. Specifically, both observational and experimental studies show that fat talk, as the most researched type of body talk, is related to thinness-oriented body dissatisfaction (e.g., body fat dissatisfaction and drive for thinness) [16, 17] and thinness-oriented disordered eating (e.g., dietary restraint and bulimia symptoms) [11, 18] in both women and men [10, 19]. Negative muscle talk, generally researched in men, has been found to be closely linked to men’s thinness-oriented body dissatisfaction and thinness-oriented disordered eating [20, 21], as well as men’s muscularity-oriented body dissatisfaction (e.g., muscularity dissatisfaction and muscle dysmorphia) and expectations of steroid use [16, 20]. Notably, although social and media pressures for being muscular have been increasing in women [22, 23], research evidence on muscle talk in women is still very limited. However, in the U.S. context, one recent study showed that muscle talk was closely related to higher muscularity dissatisfaction in women [24].

Positive body talk has gained research popularity in recent years. Related research reported that higher positive body talk was related to lower body fat dissatisfaction in girls from the U.S. [12] and higher body esteem in both boys and girls in a large-scale survey from multiple countries (i.e., Austria, Belgium, Spain, and South Korea) [25]. Moreover, in a sample of Belgian adolescent boys and girls, positive body talk was found to play a protective role in relation to lower thin/athletic-ideal internalization [26]. However, there are mixed findings on the relation between positive body talk and thinness-oriented disordered eating. For example, in a sample of French Canadian adolescent girls, Barbeau and colleagues [27] reported a significant positive relationship between positive body talk and thinness-oriented disordered eating, but there are also studies that identified a non-significant relationship between positive body talk and thinness-oriented disordered eating in both adolescent girls [12] and young adult women [13].

With samples of adult women and men from the U.S., Lin and colleagues [24] found that positive body talk in women was negatively related to women’s body fat dissatisfaction but positively associated with women’s muscularity dissatisfaction and body appreciation. However, positive body talk in men was not related to either body fat dissatisfaction or muscularity dissatisfaction but was positively related to body appreciation. Moreover, multiple linear regression analyses revealed that positive body talk was uniquely associated with higher thinness-oriented disordered eating in women and higher muscle dysmorphia symptoms in men. Taken together, these mixed findings suggest more research is needed to clarify the link between positive body talk and eating and body image disturbances.

### Body talk in the Chinese context

In China, research on body talk is still very limited [28]. Nevertheless, there has been preliminary evidence from both observational [29, 30] and experimental [28] studies supporting the positive link between body fat talk and body dissatisfaction in college women and men. Furthermore, there are also studies that explored negative body talk on social networking sites (SNSs), finding that negative body talk on SNSs was related to higher body surveillance, higher body shame, and higher cosmetic surgery consideration in both Chinese college men and women [31, 32]. However, prior studies conducted in China are confined to negative body talk, especially fat talk in women. Thus, relevant findings cannot be directly generalized to men. Also, much still remains unknown about muscle talk and positive body talk for both women and men in the Chinese context.

Furthermore, considering the perspectives of collectivism and individualism, East Asians living in societies that value collectivism orientations (e.g., conform to group standards and social norms) may be more vulnerable to other’s opinions of their physical appearance [33] than people from individualistic cultures. Although not specific to the Chinese context, Sladek and colleagues [34] showed with samples from the U.S. that Asian women had significantly more negative body talk than their White and Latina peers, and Asian and Latino men had significantly more fat talk than their White peers. Thus, it is important to conduct further research on body talk to better understand the etiology of body image and disordered eating in China.

### Measures of body talk

To date, there are several widely used measures for assessing body talk, such as the Fat Talk Scale [35], the Negative Body Talk Scale (NBTS) [36], and the Fat Talk Questionnaire (FTQ) [37] for assessing women’s negative body fat talk; the Male Body Talk Scale (MBTS) [15] for assessing men’s negative body fat and muscle talk; and the Body Talk Scale (BTS) [24] for assessing both women’s and men’s negative body fat, negative muscle talk, and positive body talk. The present study chose to validate the BTS because the BTS covers three major types of body talk (i.e., negative fat talk, negative muscle talk, and positive body talk), it can be administered to both women and men, and it has demonstrated good psychometric properties in both women and men (e.g., strong internal consistency reliability, test–retest reliability, and good concurrent validity and incremental validity) [24].

### The present study

Given the scarcity of the literature on body talk, especially muscle talk and positive body talk in Chinese adults, the present study adapted the Body Talk Scale (BTS) into Mandarin Chinese (i.e., C-BTS) and examined its psychometric properties among adult women and men. According to the findings of the parent study regarding the factor structure and psychometric properties of the BTS [24], as well as previous literature on the associations between body talk, body image, and disordered eating [10, 19–21, 28–30], we hypothesized that: (1) the C-BTS would present three factors (i.e., Negative Fat Talk, Negative Muscle Talk, and Positive Body Talk) in both women and men; (2) the three factors of the C-BTS would have adequate internal consistency reliability and test–retest reliability in both women and men; (3) the three factors of the C-BTS would demonstrate construct validity via significant relationships with other negative body talk scales, as well as relevant body image and disordered eating constructs (i.e., body fat dissatisfaction, drive for muscularity, body appreciation, functionality appreciation, body image flexibility, thinness-oriented disordered eating, and muscularity-oriented disordered eating) in both women and men; and (4) the C-BTS would demonstrate incremental validity by describing unique variance in body image (e.g., body fat dissatisfaction) and disordered eating (e.g., thinness-oriented disordered eating) constructs, beyond other negative body talk scales and covariates. In addition, the present study also explored the measurement invariance of the C-BTS across women and men. However, since the parent study did not conduct gender invariance tests, there was no basis for us to make a hypothesis for gender invariance; thus, the measurement invariance of the C-BTS across women and men was exploratory in nature.

## Method

### Participants and procedure

The protocol of the project was approved by the Institutional Review Board of the Chinese University of Hong Kong, Shenzhen. The project used Credamo (https://www.credamo.com) to collect data. Credamo has been demonstrated as a reliable online survey platform [38]. In our online survey, we set a target sample of 600 participants with 300 men and 300 women. Eligible participants were Chinese and at least 18 years old. All participants needed to give a digital informed consent first to access the survey questionnaires. To ensure data quality, we included a voice recording question in the survey which required participants to make a recording of the phrase “*I agree with all contents in the informed consent and voluntarily participate in the survey*” and upload this recording to be validated by research assistants. Surveys without a voice recording or with an unclear voice in the recording were removed. In addition, we also used a “CAPTCHA” question and two attention checks (e.g., “*Please select ‘Strongly Disagree’ for this item*.”) in the survey. Failure on the “CAPTCHA” question or either of the two attention checks would lead to a termination of the survey by the platform. Participants who completed the survey with valid responses were paid ¥10.5 (around $1.55). In addition, to assess the two-week test–retest reliability of the C-BTS, a second survey which only contained the C-BTS was re-sent to all participants two weeks after the first survey. A total of 484 participants (250 women and 234 men) took the second survey and they were further paid ¥1 (around $0.15).

Finally, 744 participants accessed our survey. After removing 144 participants who did not complete the survey with valid responses (e.g., those who did not provide a clear voice recording or failed the “CAPTCHA” and attention checks), data from 300 women and 300 men were successfully collected. Note that Credamo does not allow users to access data from unpaid participants. Thus, there were no missing data in the present study. For women, they had a mean (*SD*) age of 29.48 (7.26) years and for men, the mean (*SD*) age was 29.36 (6.81) years. Body mass index (BMI), obtained with self-reported height and weight, had a mean (*SD*) of 20.32 (2.12) kg/${\mathrm{m}}^{2}$ and 22.62 (2.38) kg/${\mathrm{m}}^{2}$ for women and men, respectively. Table 1 describes all demographic characteristics of the sample by gender (i.e., ethnicity, education, monthly income, profession, marital status, age, and BMI).

**Table 1:** Demographic characteristics

Demographics	Women	Men
	*n* (%)/*M*(*SD*)	*n* (%)/*M*(*SD*)
Ethnicity		
Han	292 (97.3)	295 (98.3)
Minority	8 (2.7)	5 (1.7)
Education		
High school or lower	14 (4.7)	12 (4.0)
College or higher	286 (95.3)	288 (96.0)
Monthly income		
$<$ 8000 ¥	176 (58.7)	148 (49.3)
$\ge$ 8000 ¥	124 (41.3)	152 (50.7)
Profession		
Students	58 (19.3)	59 (19.7)
Non-students	242 (80.7)	241 (80.3)
Marital status		
Unmarried	109 (36.3)	124 (41.3)
Married	190 (63.3)	175 (58.3)
Divorced	1 (0.3)	1 (0.3)
Age	29.48 (7.26)	29.36 (6.81)
BMI	20.32 (2.12)	22.62 (2.38)

*M* = mean, *SD* = standard deviation, BMI = body mass index (based on self-reported height and weight)

### Measures

#### Body talk scale

The Body Talk Scale (BTS) is a 14-item self-report measure developed by Lin and colleagues [24]. The instructions for the BTS were: “*The items below refer to statements that people might say about their bodies. How often have you verbally made comments about your body such as the ones below?*” Furthermore, to ensure that the BTS measures body “talk”, per the reminders used in the NBTS [36] and the MBTS [15], in the present study, participants were further reminded that researchers were only interested in what they actually *SAY* but not what they *THINK*. Also, participants were instructed that even if they might not use these exact words, researchers were interested in whether they say similar things with the same meanings. The BTS has three subscales including the Negative Fat Talk (5 items; e.g., “*I need to lose some weight*.”), Negative Muscle Talk (5 items; e.g., “*I wish I was more muscular*.”), and Positive Body Talk (4 items; “*I like the way I look*.”) subscales. The items of the BTS are rated on a 6-point scale, from 1 (Never) to 6 (Always – several times per day). The subscale scores were calculated by averaging the scores of items in each subscale. Higher total scores on the BTS indicate higher levels of negative and positive body talk.

To obtain the Chinese version of the BTS, the English version of the BTS was first translated into Chinese (Mandarin) based on the standard guidelines from cross-cultural scale adaptation [39, 40]. Specifically, two bilingual Chinese research assistants (Chinese and English) forward-translated the BTS into Chinese independently. After a group discussion among the two translators and a third bilingual translator, the two Chinese translations were synthesized. Next, back-translation was conducted independently by two additional bilingual research assistants who had no prior knowledge of the BTS. Then, the research team, comprising all forward- and back-translators and two experts in body image, evaluated the forward- and back-translations, which resulted in a pre-final version. In the next step, 60 Chinese adults (50% men) were recruited to pilot the understandability of the items on a 5-point scale, from 1 (Do not understand at all) to 5 (Understanding completely). The mean scores of understandabilities in men and women were 4.73 (*SD* = 0.60) and 4.70 (*SD* = 0.52), respectively, with the mean scores for all items in both men and women greater than 4. As such, the pre-final version (i.e., C-BTS) was approved by the research team without any further revisions, and it was then used in the subsequent investigation.

#### Negative body talk for women and men

As in Lin and colleagues [24] and Warnick and colleagues [17], we used two gender-specific negative body talk measures that are available for women and men. Specifically, the Negative Body Talk Scale (NBTS) [36] measures women’s tendency to engage in negative body talk. The NBTS has 13 items (e.g., “*I need to go on a diet*.”), which are rated on a seven-point scale from 1 (Never) to 7 (Always). The NBTS has a total score which incorporates two subscales (i.e., body concerns and body comparisons). A total score is obtained by averaging the responses of all items, and the subscale scores are obtained by averaging the responses of the items belonging to each subscale. Higher total scores on the NBTS indicate higher levels of negative body talk. The Chinese version of the NBTS showed high internal consistency reliability ($\alpha$ = 0.92) and the scores of the NBTS were closely related to body dissatisfaction in Chinese college students (*r* = 0.46, *p* < 0.001) [30]. However, as indicated in Sladek and colleagues [34], item 13 (“*You never have to worry about gaining weight*.”) may be problematic (e.g., this item is worded differently than other items). Thus, following Sladek and colleagues [34], we removed item 13. In the present sample of women, the Cronbach’s $\alpha$ values were 0.92, 0.93, and 0.89 for the total scale, the body concerns subscale, and the body comparisons subscale, respectively.

For assessing men’s engagement in negative body talk, we used the Male Body Talk Scale (MBTS) [15]. The MBTS has 16 items (e.g., “*I want a six-pack*.”) which are rated on a seven-point scale from 1 (Never) to 7 (Always). The MBTS has two subscales, the fat talk and muscle talk subscales. The subscale scores are obtained by averaging the responses of the items belonging to each subscale. Higher total scores on the MBTS indicate higher levels of negative body talk. The MBTS showed good psychometric properties (e.g., strong internal consistency reliability and good construct validity) in previous studies [15, 34]. Since there is no Chinese version of the MBTS available, the research team translated the MBTS using identical procedures discussed for translating the BTS in the present study (see the procedures in Sect. "Body talk scale"). The mean score of understandability was 4.84 (*SD* = 0.42) for the MBTS. Thus, this version was used in the present study without further modifications. In the present sample of men, CFA for the two-factor structure showed adequate model fit, with ${\chi }^{2}$(103) = 629.35 (*p* < 0.001), CFI = 0.97, TLI = 0.97, and SRMR = 0.09, and the factor loadings of the 16 items ranged from 0.73 to 0.94. In the present sample of men, the Cronbach’s $\alpha$ values were 0.93 and 0.96 for the fat talk subscale and the muscle talk subscale, respectively.

#### Body fat dissatisfaction

The Body Dissatisfaction subscale of the Eating Disorder Inventory (EDI-BD) [41] measures negative attitudes towards one's own body weight/shape. The EDI-BD subscale has 9 items (e.g., “*I think that my stomach is too big*.”), which are rated on a six-point scale ranging from 1 (Never) to 6 (Always). Five items were reverse-coded. A total score can be calculated by summing the responses of the items. Higher total scores on the EDI-BD indicate higher levels of body fat dissatisfaction. The Chinese version of the EDI showed good internal consistency reliability and good convergent and discriminant validity in both clinical and non-clinical Chinese populations [42–44]. In the present study, the EDI-BD had Cronbach’s $\alpha$ values of 0.93 and 0.92 for women and men, respectively.

#### Drive for muscularity for women and men

The Female Muscularity Scale (FMS) [22] measures drive for muscularity (i.e., the desire to be more muscular and the behaviors to enhance muscularity) in women. The FMS includes 10 items (e.g., “*I wish I were more toned*.”), which are responded to on a five-point scale with response options from 1 (Never) to 5 (Always). A total score can be calculated by averaging the responses of the items. Higher total scores represent higher levels of drive for muscularity. The Chinese version of the FMS showed good reliability (i.e., internal consistency reliability) and good validity (i.e., convergent validity and incremental validity) in Chinese women [23]. In the present sample of women, the FMS had a Cronbach’s α of 0.91.

The Drive for Muscularity Scale (DMS) [45] measures drive for muscularity in men. The DMS has 15 items (e.g., “*I wish that I were more muscular*.”), which are rated on a six-point scale from 1 (Always) to 6 (Never). A total score can be calculated by averaging the reverse-coded responses of the items. Higher total scores represent higher levels of drive for muscularity. The Chinese version of the DMS showed good internal consistency reliability and good convergent validity in Chinese adult men [46]. In the present sample of men, the DMS had a Cronbach’s α value of 0.90.

#### Thinness-oriented disordered eating

The 12-item short form of the Eating Disorder Examination-Questionnaire (EDE-QS) [47] measures maladaptive eating patterns driven by the pursuit of thinness. The EDE-QS contains 12 items (e.g., “*Have you been deliberately trying to limit the amount of food you eat to influence your weight or shape (whether or not you have succeeded)*?”) with response options ranging from 0 (0 days/not at all) to 3 (6–7 days/markedly). A total score can be calculated by summing the responses of the items. Higher total scores indicate higher levels of thinness-oriented disordered eating. The Chinese version of the EDE-QS had good psychometric properties (i.e., high internal consistency reliability and good convergent validity) in Chinese adults [48]. In the present study, the EDE-QS had Cronbach’s α values of 0.88 and 0.86 for women and men, respectively.

#### Muscularity‐oriented disordered eating

The 15-item Muscularity-Oriented Eating Test (MOET) [49] measures maladaptive eating patterns driven by the pursuit of muscularity. Items of the MOET (e.g., “*I have continued eating despite feeling full, in attempting to influence my muscularity*.”) are rated on a five-point scale from 0 (Never true) to 4 (Always true). A total score can be calculated by averaging the responses of the items. Higher total scores indicate higher levels of muscularity-oriented disordered eating. The Chinese version of the MOET showed good psychometric properties (e.g., high internal consistency reliability and good convergent validity) in Chinese adult women and men [46, 50]. In the present study, the MOET had a Cronbach’s α of 0.90 and 0.91 for women and men, respectively.

#### Body appreciation

The Body Appreciation Scale-2 (BAS-2) [51] measures the extent to which individuals accept, respect, and cherish their bodies. The BAS-2 has 10 items (e.g., “*I feel good about my body*.”), which are rated on a five-point scale from 1 (Never) to 5 (Always). A total score can be calculated by averaging the responses of the items. Higher total scores of the BAS-2 indicate higher levels of body appreciation. The Chinese version of the BAS-2 showed good internal consistency reliability and good convergent validity in Chinese adults [52]. In the present study, the BAS-2 had Cronbach’s α values of 0.92 and 0.90 for women and men, respectively.

#### Functionality appreciation

The Functionality Appreciation Scale (FAS) [53] measures the extent to which individuals appreciate what their bodies can do. The FAS has 7 items (e.g., “*I appreciate my body for what it is capable of doing*.”), which are rated on a five-point scale from 1 (Strongly disagree) to 5 (Strongly agree). A total score can be calculated by averaging the responses of the items. Higher total scores on the FAS indicate higher levels of functionality appreciation. The Chinese version of the FAS showed sound psychometric properties (e.g., high internal consistency reliability and good convergent validity) in Chinese adults [54, 55]. The FAS had Cronbach’s α values of 0.77 and 0.78 for women and men in the present study.

#### Body image flexibility

The 5-item Body Image-Acceptance and Action Questionnaire (BI-AAQ-5) [56] assesses the extent to which individuals can accept, but not try to avoid or change, negative thoughts or feelings about their bodies. The items of the BI-AAQ-5 (e.g., “*Worrying about my weight makes it difficult for me to live a life that I value*.”) are rated on a seven-point scale from 1 (Never true) to 7 (Always true). All items were reverse-coded. A total score can be calculated by averaging the reverse-coded responses of the items. Higher total scores indicate higher levels of body image flexibility. The Chinese version of the BI-AAQ-5 showed sound psychometric properties (e.g., high internal consistency reliability and adequate convergent validity) in Chinese adults [57]. In the present study, the BI-AAQ-5 had Cronbach’s α values of 0.90 and 0.85 for women and men, respectively.

### Statistical analysis

Data analyses in the present study were conducted via the *psych* [58] and *lavaan* [59] packages on *R* version 4.2.0 [60]. Given the prominence of gender differences in body talk [25, 61, 62], data analyses were conducted separately for women and men. As recommended in Swami and Barron [40], the EFA-to-CFA approach was used to examine the factor structure of the BTS in the Chinese context. Specifically, the total sample of women (*N* = 300) was randomly split into two equal subsamples, one for exploratory factor analysis (EFA; *n* = 150) and the other for confirmatory factor analysis (CFA; *n* = 150). Similarly, the sample of men (*N* = 300) was also split into two equal subsamples, one for EFA (*n* = 150) and the other for CFA (*n* = 150).

For EFA, Oblimin rotation and the ordinary least squares estimator were used [63]. To determine the number of factors, parallel analysis was used [64]. Based on the commonly used participant-to-item ratio of 10:1 for the minimum sample size for EFA [65], the minimum number of participants was 140; thus, the current sample sizes of 150 for EFA for both women and men were adequate. Moreover, according to the post-hoc results regarding the factor loadings of EFA (all above 0.40), our sample sizes of 150 for both women and men were also adequate [66]. As recommended in Ferguson and Cox [67], factor loadings of 0.40 are considered adequate, whereas an item with loadings of 0.40 or higher on two or more factors is a cross-loading item which may be problematic and can be deleted.

For CFA, by using an online calculator for estimating the minimum sample size for structural equation modeling [68], with conditions of a moderate effect size of 0.30, power of 0.80, 3 latent variables, 14 observed variables, and probability level of 0.05, the recommended sample size was 119. Thus, the sample sizes of 150 for CFA for both women and men was adequate. The mean- and variance-adjusted weighted least squares estimator (WLSMV) were used for model estimation [69]. To evaluate the model fit of CFA, we used comparative fit index (CFI), Tucker-Lewis Index (TLI), and standardized root mean square residual (SRMR). Based on Hu and Bentler [70]: both CFI and TLI greater or equal to 0.95 indicates a good fit while greater than or equal to 0.90 indicates an acceptable fit; and SRMR less than or equal to 0.06 indicates a good fit while SRMR less than or equal to 0.08 indicates an acceptable fit. In addition, as reported in Shi and colleagues [71], because RMSEA tended to reject fitted models for ordinal data, RMSEA was not reported in the present study. In addition, to examine measurement invariance across women and men, we conducted the stepwise measurement invariance tests to evaluate configural, metric, and scalar models [72]. Based on Cheung and Rensvold [73], CFI < 0.010 and SRMR < 0.030 indicated measurement invariance.

To evaluate internal consistency reliability of the BTS, we reported the Cronbach’s $\alpha$ [74] and McDonald’s $\omega$ [75] for ordinal data. To evaluate the test–retest reliability of the BTS, we reported the intraclass coefficient (ICC). For internal consistency reliability, values above 0.70 are adequate [76]. For ICC, values between 0.50 and 0.75 are moderate and values above 0.75 are good [77]. Finally, Pearson correlations were calculated to examine the convergent and concurrent validity of the BTS. Hierarchical regressions were used to evaluate the incremental validity of the BTS when other measures of negative body talk (i.e., the NBTS for women and the MBTS for men) and covariates (i.e., age and BMI) were controlled.

## Results

### Exploratory factor analysis

For women, the results of Kaiser–Meyer–Olkin (KMO) measure of sampling adequacy (KMO = 0.87) and Bartlett’s test of sphericity [${\chi }^{2}$(91) = 1480.79, *p* < 0.001] suggested the appropriability of these data for EFA. As shown in Additional file 1: Fig. S1, parallel analysis (${\lambda }_{1}$= 4.86 > 0.71, ${\lambda }_{2}$= 2.39 > 0.45, ${\lambda }_{3}$= 1.36 > 0.33, and ${\lambda }_{4}$=  −  0.05 < 0.24) indicated three factors, which could account for 73.42% of the total variance. The factor loadings of EFA on the intended factors ranged from 0.73 to 0.92 (see Table 2).

**Table 2:** Factor loadings of the exploratory factor analyses for women and men

Item	Women (*n* = 150)			Men (*n* = 150)		
	Negative fat talk	Positive body talk	Negative muscle talk	Negative fat talk	Positive body talk	Negative muscle talk
1. I need to lose some weight	**.91**	− .01	− .04	**.92**	.01	− .13
2. I feel fat	**.87**	− .11	− .08	**.90**	− .16	− .07
3. My clothes are too tight	**.79**	− .10	− .02	**.67**	− .05	.06
4. I should stop eating fattening foods	**.73**	.00	.02	**.81**	.01	.15
5. I need to exercise more so I can lose some weight	**.82**	.07	.15	**.90**	.09	.08
6. I wish I was more muscular	− .09	− .14	**.92**	.08	− .07	**.82**
7. I wish my body were stronger	− .03	.04	**.89**	− .04	− .02	**.84**
8. I should eat foods that promote muscle growth	.01	− .02	**.85**	− .13	− .05	**.85**
9. I need to lift weights more often to build muscle	.14	.15	**.82**	.16	.09	**.77**
10. I like the way I look	− .15	**.82**	− .05	− .08	**.78**	− .07
11. I feel good about my body	− .21	**.78**	.04	.01	**.89**	− .12
12. I am proud of what my body can do	− .02	**.83**	.02	− .05	**.78**	.04
13. I am happy with my eating habits	− .08	**.78**	− .07	− .08	**.76**	− .06
14. I am satisfied with my exercise habits	.23	**.85**	.04	.08	**.84**	.16
Inter-factor correlation matrix						
Negative fat talk	1			1		
Positive body talk	− .43	1		− .36	1	
Negative muscle talk	.08	.09	1	.18	− .18	1

Factor loadings in bold are loadings greater than .40

For men, the results of Kaiser–Meyer–Olkin (KMO) measure of sampling adequacy (KMO = 0.87) and Bartlett’s test of sphericity [${\chi }^{2}$(91) = 1353.50, *p* < 0.001] also suggested the appropriability for EFA. As shown in Additional file 1: Fig. S2, parallel analysis (${\lambda }_{1}$= 4.84 > 0.69, ${\lambda }_{2}$= 1.82 > 0.43, ${\lambda }_{3}$= 1.67 > 0.34, and ${\lambda }_{4}$= − 0.04 < 0.26) indicated three factors, which could account for 71.06% of the total variance. The factor loadings of EFA on the intended factors ranged from 0.67 to 0.92 (see Table 2).

### Confirmatory factor analysis

For women, results of the CFA showed that the three-factor structure had adequate model fit, with ${\chi }^{2}$(74) = 199.04 (*p* < 0.001), CFI = 0.96, TLI = 0.95, and SRMR = 0.08. For men, the CFA results also showed adequate model fit for the three-factor structure, with ${\chi }^{2}$(74) = 149.64 (*p* < 0.001), CFI = 0.98, TLI = 0.98, and SRMR = 0.07. The factor loadings for women (a range of 0.71–0.97) and men (a range of 0.75–0.98) are described in Table 3.

**Table 3:** Factor loadings of the confirmatory factor analyses for women and men

Item	Women (*n* = 150)			Men (*n* = 150)		
	Negative fat talk	Positive body talk	Negative muscle talk	Negative fat talk	Positive body talk	Negative muscle talk
1. I need to lose some weight	.86			.98		
2. I feel fat	.97			.91		
3. My clothes are too tight	.71			.76		
4. I should stop eating fattening foods	.75			.77		
5. I need to exercise more so I can lose some weight	.75			.84		
6. I wish I was more muscular			.86			.92
7. I wish my body were stronger			.85			.87
8. I should eat foods that promote muscle growth			.84			.75
9. I need to lift weights more often to build muscle			.85			.75
10. I like the way I look		.88			.90	
11. I feel good about my body		.91			.94	
12. I am proud of what my body can do		.84			.76	
13. I am happy with my eating habits		.81			.87	
14. I am satisfied with my exercise habits		.74			.77	
Inter-factor correlation matrix						
Negative fat talk	1			1		
Positive body talk	− .58***	1		− .57***	1	
Negative muscle talk	− .03	.07	1	− .16*	.003	1

*p < .05, ***p < .001

### Gender invariance and differences

As shown in Table 4, placing constraints subsequently on configuration, factor loadings and intercepts did not lead to a worsening of model fit, suggesting that a scalar invariance across women and men was supported for the C-BTS. Independent *t* tests showed that there was significantly more frequent fat talk in women (*M* = 3.19, *SD* = 1.15) than men (*M* = 2.78, *SD* = 1.16),* t*(598) = 4.43 (*p* < 0.001; *d* = 0.35), and there was significantly less frequent muscle talk in women (*M* = 2.40, *SD* = 1.15) than men (*M* = 3.65, *SD* = 1.10), *t*(598) = 13.67 (*p* < 0.001; *d* = 1.11). However, there were no significant differences between women (*M* = 3.50, *SD* = 1.15) and men (*M* = 3.46, *SD* = 1.09) in terms of the frequency of positive body talk, *t*(598) = 0.45 (*p* = 0.651; *d* = 0.04).

**Table 4:** Measurement invariance across women and men

	${\chi }^{2}$	*df*	CFI	TLI	SRMR	$\Delta$ CFI	$\Delta$ SRMR
Women (*N* = 300)	290.58***	74	0.968	0.961	0.073		
Men (*N* = 300)	240.26***	74	0.981	0.976	0.065		
Configural model	528.92***	148	0.975	0.970	0.069		
Metric model	551.42***	159	0.974	0.971	0.071	− 0.001	0.002
Scalar model	661.56***	212	0.971	0.975	0.069	− 0.003	− 0.002

***p < .001

### Reliability

For women, the Cronbach’s $\alpha$ values were 0.91, 0.92, and 0.92 for the Negative Fat Talk, Negative Muscle Talk, and Positive Body Talk subscales, respectively. The McDonald’s $\omega$ values were 0.91, 0.90, and 0.91 for the Negative Fat Talk, Negative Muscle Talk, and Positive Body Talk subscales, respectively. Furthermore, the two-week ICC values were 0.78, 0.85, and 0.80 for the Negative Fat Talk, Negative Muscle Talk, and Positive Body Talk subscales, respectively. Thus, all three subscales had good internal consistency reliability and good test–retest reliability for women.

For men, the Cronbach’s $\alpha$ values were 0.93, 0.89, and 0.92 for the Negative Fat Talk, Negative Muscle Talk, and Positive Body Talk subscales, respectively. The McDonald’s $\omega$ values were 0.92, 0.88, and 0.90 for the Negative Fat Talk, Negative Muscle Talk, and Positive Body Talk subscales, respectively. Furthermore, the two-week ICC values were 0.79, 0.68, and 0.80 for the Negative Fat Talk, Negative Muscle Talk, and Positive Body Talk subscales, respectively. Thus, for men, all three subscales had good internal consistency reliability, the Negative Fat Talk and Positive Body Talk subscales had good test–retest reliability, and the Negative Muscle Talk scale had moderate test–retest reliability.

### Convergent and concurrent validity

For women, as described in Table 5, the Negative Fat Talk subscale scores correlated significantly with the NBTS total scores (*r* = 0.74, *p* < 0.001), suggesting good convergent validity of the Negative Fat Talk subscale. Furthermore, each subscale of the C-BTS also had significant correlations with corresponding eating and body image outcomes (e.g., the Negative Fat Talk subscale and body fat dissatisfaction, *r* = 0.67, *p* < 0.001; the Negative Muscle Talk subscale and drive for muscularity, *r* = 0.66, *p* < 0.001; and the Positive Body Talk subscale and body appreciation, *r* = 0.75, *p* < 0.001), suggesting strong concurrent validity of the C-BTS in women.

**Table 5:** Bivariate correlations between the Chinese-Body Talk Scale and related variables for women (*N* = 300)

Variables	1	2	3	4	5	6	7	8	9	10	11	12	13	14	15
1. C-BTS-Negative fat talk	1														
2. C-BTS-Negative muscle talk	.03	1													
3. C-BTS-Positive body talk	− .45***	.08	1												
4. NBTS-Total	.74***	.08	− .51***	1											
5. NBTS-Body concerns	.82***	− .10	− .57***	.90***	1										
6. NBTS-Body comparisons	.45***	.28***	− .31***	.86***	.56***	1									
7. EDI-BD-Body fat dissatisfaction	.67***	− .02	− .73***	.70***	.77***	.45***	1								
8. FMS-Drive for muscularity	.22***	.66***	− .01	.34***	.15**	.47***	.14*	1							
9. EDE-QS-Thinness-oriented disordered eating	.62***	.00	− .38***	.68***	.67***	.51***	.59***	.23***	1						
10. MOET-Muscularity-oriented disordered eating	.50***	.28***	− .16**	.59***	.50***	.55***	.34***	.49***	.70***	1					
11. BAS-2-Body appreciation	− .46***	− .02	.75***	− .57***	− .57***	− .42***	− .73***	− .07	− .55***	− .30***	1				
12. FAS-Functionality appreciation	− .19***	.07	.36***	− .24***	− .25***	− .16**	− .35***	.03	− .22***	− .12*	.49***	1			
13. BI-AAQ-Body image flexibility	− .56***	− .01	.46***	− .66***	− .65***	− .50***	− .62***	− .16**	− .69***	− .58***	.59***	.34***	1		
14. Age	.03	.05	.06	− .01	− .002	− .01	− .02	.06	− .05	.02	.09	.06	.10	1	
15. BMI	.51***	.01	− .32***	.35***	.47***	.12*	.55***	.02	.32***	.14*	.34***	− .15*	− .35***	.24***	1
*Mean*	3.19	2.40	3.50	4.08	3.89	4.28	30.91	3.01	9.84	1.40	3.64	4.31	5.12	29.48	20.32
*SD*	1.15	1.15	1.15	1.18	1.44	1.22	10.92	0.80	5.99	0.70	0.70	0.44	1.28	7.26	2.12

C-BTS = Chinese-Body Talk Scale, NBTS = Negative Body Talk Scale, EDI-BD = Body Dissatisfaction subscale of the Eating Disorder Inventory, FMS = Female Muscularity Scale, EDE-QS = Short Form of the Eating Disorder Examination-Questionnaire, MOET = Muscularity-Oriented Eating Test, BAS-2 = Body Appreciation Scale-2, BI-AAQ = Body Image Acceptance and Action Questionnaire, BMI = body mass index

*p < .05; **p < .01; ***p < .001

The correlation patterns in men are described in Table 6. Specifically, the Negative Fat Talk subscale scores correlated significantly with the MBTS Fat Talk subscale scores (*r* = 0.84, *p* < 0.001), suggesting its good convergent validity. The Negative Muscle Talk subscale scores had a significant correlation with the MBTS Muscle Talk subscale scores (*r* = 0.77, *p* < 0.001), suggesting its good convergent validity. Furthermore, like in women, each subscale of the C-BTS also had significant correlations with corresponding eating and body image outcomes (e.g., the Negative Fat Talk subscale and body fat dissatisfaction, *r* = 0.72, *p* < 0.001; the Negative Muscle Talk subscale and drive for muscularity, *r* = 0.79, *p* < 0.001; and the Positive Body Talk subscale and body appreciation, *r* = 0.75, *p* < 0.001), suggesting strong concurrent validity of the C-BTS in men.

**Table 6:** Bivariate correlations between the Chinese-Body Talk Scale and related variables for men (*N* = 300)

Variables	1	2	3	4	5	6	7	8	9	10	11	12	13	14
1. C-BTS-Negative fat talk	1													
2. C-BTS-Negative muscle talk	.17**	1												
3. C-BTS-Positive body talk	− .45***	− .08	1											
4. MBTS-Fat talk	.84***	.23***	− .42***	1										
5. MBTS-Muscle talk	.10	.77***	.01	.23***	1									
6. EDI-BD-Body fat dissatisfaction	.72***	.14**	− .72***	.71***	.03	1								
7. DMS-Drive for muscularity	.19**	.79***	− .14*	.30***	.79***	.18**	1							
8. EDE-QS-Thinness-oriented disordered eating	.73***	.29***	− .44***	.74***	.27***	.68***	.41***	1						
9. MOET-Muscularity-oriented disordered eating	.54***	.49***	− .25***	.64***	.53***	.45***	.65***	.69***	1					
10. BAS-2-Body appreciation	− .45***	− .09	.75***	− .41***	.03	− .74***	− .14*	− .53***	− .31***	1				
11. FAS-Functionality appreciation	− .11	− .04	.34***	− .07	.11	− .30***	− .03	− .25***	− .09	.47***	1			
12. BI-AAQ-Body image flexibility	− .61***	− .31***	.43***	− .62***	− .33***	− .62***	− .44***	− .75***	− .71***	.52***	.21***	1		
13. Age	.12*	− .08	.10	.06	− .04	− .07	− .06	.01	.05	.18**	.11	− .001	1	
14. BMI	.55***	− .12*	− .20***	.50***	− .16**	.42***	− .10	.41***	.22***	− .20***	− .02	− .30***	.31***	1
*Mean*	2.78	3.65	3.46	3.65	4.52	28.56	3.11	8.50	1.38	3.61	4.28	5.10	29.36	22.62
*SD*	1.16	1.10	1.09	1.58	1.30	9.97	0.82	5.66	0.71	0.67	0.43	1.15	6.81	2.38

C-BTS = Chinese-Body Talk Scale, NBTS = Negative Body Talk Scale, EDI-BD = Body Dissatisfaction subscale of the Eating Disorder Inventory, DMS = Drive for Muscularity Scale, EDE-QS = Short Form of the Eating Disorder Examination-Questionnaire, MOET = Muscularity-Oriented Eating Test, BAS-2 = Body Appreciation Scale-2, BI-AAQ = Body Image Acceptance and Action Questionnaire. BMI = body mass index

*p < .05, **p < .01, ***p < .001

### Incremental validity

As presented in Additional file 1: Table S1, for women, when adjusting for covariates (i.e., age and BMI) and the NBTS scores, hierarchical linear regressions revealed that except for body image flexibility (${\Delta R}_{adj}^{2}$ = 0.01, *p* = 0.054), the C-BTS subscale scores explained additional, unique variance in all other potential body image and disordered eating outcomes, namely body fat dissatisfaction (${\Delta R}_{adj}^{2}$ = 0.15, *p* < 0.001), drive for muscularity (${\Delta R}_{adj}^{2}$ = 0.40, *p* < 0.001), thinness-oriented disordered eating (${\Delta R}_{adj}^{2}$ = 0.03, *p* = 0.002), muscularity-oriented disordered eating (${\Delta R}_{adj}^{2}$ = 0.09, *p* < 0.001), body appreciation (${\Delta R}_{adj}^{2}$ = 0.27, *p* < 0.001), and functionality appreciation (${\Delta R}_{adj}^{2}$ = 0.07, *p* < 0.001). These findings suggest the incremental validity of the C-BTS in women.

For men, the hierarchical linear regressions (Additional file 1: Table S2) showed that when adjusting for covariates (i.e., age and BMI) and the MBTS scores, the BTS subscale scores explained additional, unique variance in all potential body image and disordered eating outcomes, including body fat dissatisfaction (${\Delta R}_{adj}^{2}$ = 0.20, *p* < 0.001), drive for muscularity (${\Delta R}_{adj}^{2}$ = 0.08, *p* < 0.001), thinness-oriented disordered eating (${\Delta R}_{adj}^{2}$ = 0.06, *p* < 0.001), muscularity-oriented disordered eating (${\Delta R}_{adj}^{2}$ = 0.01, *p* = 0.022), body appreciation (${\Delta R}_{adj}^{2}$ = 0.37, *p* < 0.001), functionality appreciation (${\Delta R}_{adj}^{2}$ = 0.12, *p* < 0.001), and body image flexibility (${\Delta R}_{adj}^{2}$ = 0.07, *p* < 0.001). These findings suggest the incremental validity of the C-BTS in men.

## Discussion

The present study adapted a Chinese version of the BTS (i.e., C-BTS) and evaluated its psychometric properties in Chinese adult women and men. Results replicated the three-factor structure of the BTS [24] and further identified measurement invariance of the three-factor structure across women and men. Moreover, results also revealed that the C-BTS had good reliability and validity.

Interestingly, analyses of gender differences in the scores on the C-BTS showed that women engaged in more frequent fat talk and less frequent muscle talk than men, but women and men showed similar frequencies of positive body talk. These findings on fat talk and muscle talk converge with evidence indicating gender differences in socially promoted body ideals in the Chinese context, with an emphasis of thinness in women and muscularity in men, especially for young Chinese adults [78, 79]. However, the findings are inconsistent with the parent study conducted in the U.S. which showed no gender differences in either fat talk or muscle talk and a marginally significant gender difference in positive body talk, with men engaging in positive body talk more frequently than women [24]. This inconsistency may be partly due to cultural differences observed across collectivism and individualism [25], with China as a highly collectivistic culture and the U.S. as a highly individualistic culture [80, 81]. Specifically, in a collectivistic culture, Chinese people value embeddedness in group relations, conformity to the group standards, and reliance on social norms to guide social behaviors, all of which may augment social pressures on body image and increase body comparisons for both women and men [82]. Thus, under the influences of conforming to the socially-promoted body ideals of thinness for women and muscularity for men, Chinese women may talk more about body fat, whereas Chinese men may talk more about muscles. However, regarding the non-significant gender difference in positive body talk, to the best of our knowledge, there are no studies on positive body talk in the Chinese context. Thus, we did not have any prior expectations (i.e., research questions were exploratory in nature) that may help explain this finding. Though speculative in nature, it may be the case that because of the aforementioned pressures to conform to body image ideals expected of women and men, positive body talk may be less part of the contemporary Chinese “zeitgeist” as it is in Western cultures. Continued research is needed to confirm and explain this non-significant finding in the Chinese context. Furthermore, it would also be interesting to explore whether the patterns of gender differences in the BTS subscales can be replicated in other collectivistic nations (e.g., Japan and South Korea), findings that may help elucidate the potential influence of collectivism vs. individualism on gender differences in body talk.

Regarding the reliability of the C-BTS, the internal consistency coefficients of the three subscales were adequate ($\alpha \mathrm{s}$ and ωs ≥ 0.88 in all analyses) for both women and men. Good test–retest reliability of the three subscales (ICC values ≥ 0.78) was identified in women. For men, good test–retest reliability (ICC values ≥ 0.79) was identified for the Negative Fat Talk and Positive Body Talk subscales, and moderate test–retest reliability (ICC values = 0.68) was identified for the Negative Muscle Talk subscale. These findings are consistent with the parent study which also revealed adequate internal consistency reliability for all subscales, and relatively lower test–retest reliability for the Negative Muscle Talk subscale than the other two subscales [24]. In addition, the moderate test–retest reliability for the Negative Muscle Talk subscale was also consistent with previous research examining the test–retest reliability of the Muscle Talk subscale on the MBTS in men [15]. It should be noted that negative muscle talk had lower test–retest reliability compared to other subscales in men, which may be because body talk for men is likely influenced by contextual factors (e.g., body talk is more likely to happen in certain contexts for men such as at the gym [14, 82]). Thus, these contextual factors might contribute to the diminished temporal stability of the Negative Muscle Talk subscale in men as the present sample of men might not have been in the same contexts (e.g., at the gym) across the two surveys. Overall, these findings indicate the scores on the C-BTS are reliable and have moderate-to-good temporal stability when used in Chinese women and men.

Regarding the validity of the C-BTS, similar to the parent study [24], good convergent and concurrent validity of the C-BTS for both women and men were supported via significant correlations between the C-BTS subscales scores and the negative body talk scales as well as measures of body image and disordered eating. In addition, also consistent with the parent study [24], good incremental validity of the C-BTS for both women and men was supported as the C-BTS scores explained unique variance in body image and disordered eating outcomes, beyond existing measures of negative body talk and covariates. These findings suggest that body talk as measured by the C-BTS may be uniquely associated with body image and disordered eating outcomes for both Chinese men and women. Thus, it may be useful to include body talk to inform prevention and intervention strategies of body image and disordered eating (e.g., the session of "Banish Body Talk" in the "Dove Confident Me" intervention [84]) in Chinese adults, although future research is needed to validate the temporal relationships between body talk and body image and disordered eating outcomes.

### Strengths, limitations and future directions

A major strength of the present study includes the introduction of the BTS in a non-Western context with robust cross-cultural measurement adaption procedures. Furthermore, the present study also used recommended approaches (e.g., the EFA-to-CFA approach [40]) for validating body image instruments and comprehensively assessing the psychometric properties of the C-BTS in adult Chinese women and men. Strengths aside, there are limitations and related future directions. First, the adult samples were recruited online, which might have contributed to the majority of participants in our sample having an education level of college or above and/or an average BMI lower than the average BMI observed in nationally representative surveys in China (e.g., around 24 kg/m^2^ [85]). Thus, the sample in the present study might not be able to fully represent the whole Chinese adult population. Second, the findings from non-clinical adults in the present study may not generalize to Chinese adolescents and clinical patients (e.g., individuals with eating disorders or body dysmorphia), and further validation of the C-BTS in these Chinese populations is needed. Relatedly, considering potential cultural differences in body talk (e.g., [25]), future studies may also examine the cross-cultural invariance of and differences in the BTS (e.g., China vs. U.S.).

## Conclusion

In summary, the results of the present study support the three-factor structure and good psychometric properties of a Chinese adaptation of the BTS in women and men from China. Our study provides a valuable tool for future studies to explore the causes and health consequences of three types of body talk, including fat talk, muscle talk, and positive body talk in the Chinese context.

### Supplementary Information

**Additional file 1: Figure S1** Parallel analysis scree plot for women. **Figure S2** Parallel analysis scree plot for men. **Table S1** Hierarchical linear regressions using the Chinese-Body Talk Scale to describe unique variance in body dissatisfaction and disordered eating in women (*N* = 300). **Table S2** Hierarchical linear regressions using the Chinese-Body Talk Scale to describe unique variance in body dissatisfaction and disordered eating in men (*N* = 300)

## Data Availability

The data are available from the corresponding author upon reasonable request.

## Abbreviations

BAS-2: Body Appreciation Scale-2
BI-AAQ-5: The 5-item Body Image-Acceptance and Action Questionnaire
BMI: Body Mass Index
BTS: Body Talk Scale
C-BTS: Chinese version of the Body Talk Scale
CFA: Confirmatory factor analyses
C-FAS: Chinese version of the Functionality Appreciation Scale
CFI: Comparative Fit Index
DMS: Drive for Muscularity Scale
EDE-QS: 12-Item Short form of the Eating Disorder Examination Questionnaire
EDI: Eating Disorder Inventory
EDI-BD: Body Dissatisfaction subscale of the Eating Disorder Inventory
EDs: Eating disorders
EFA: Exploratory factor analyses
FAS: Functionality Appreciation Scale
FMS: Female Muscularity Scale
FTQ: Fat Talk Questionnaire
ICC: Intraclass coefficient
KMO: Kaiser–Meyer–Olkin
MBTS: Male Body Talk Scale
MCAR: Missing completely at random
MOET: Muscularity-oriented eating test
NBTS: Negative Body Talk Scale
RMSEA: Root mean square error of approximation
SNSs: Social networking sites
SRMR: Standardized root mean square residual
TLI: Tucker–Lewis Index
WLSMV: Mean- and variance-adjusted weighted least squares estimator
