# Supplementary material for: Translation and validation of a Chinese version of the body talk scale for women and men
Source: J Eat Disord. 2023 Sep 11;11:153. doi: 10.1186/s40337-023-00884-y (PMC10494420; doi:10.1186/s40337-023-00884-y)
Supplement: Supplementary file 1 — Additional file 1: Figure S1 Parallel analysis scree plot for women. Figure S2 Parallel analysis scree plot for men. Table S1 Hierarchical linear regressions using the Chinese-Body Talk Scale to describe unique variance in body dissatisfaction and disordered eating in women (N = 300). Table S2 Hierarchical linear regressions using the Chinese-Body Talk Scale to describe unique variance in body dissatisfaction and disordered eating in men (N = 300) [file 40337_2023_884_MOESM1_ESM.docx]

**Supplementary Materials**

Figure S1. Parallel analysis scree plot for women


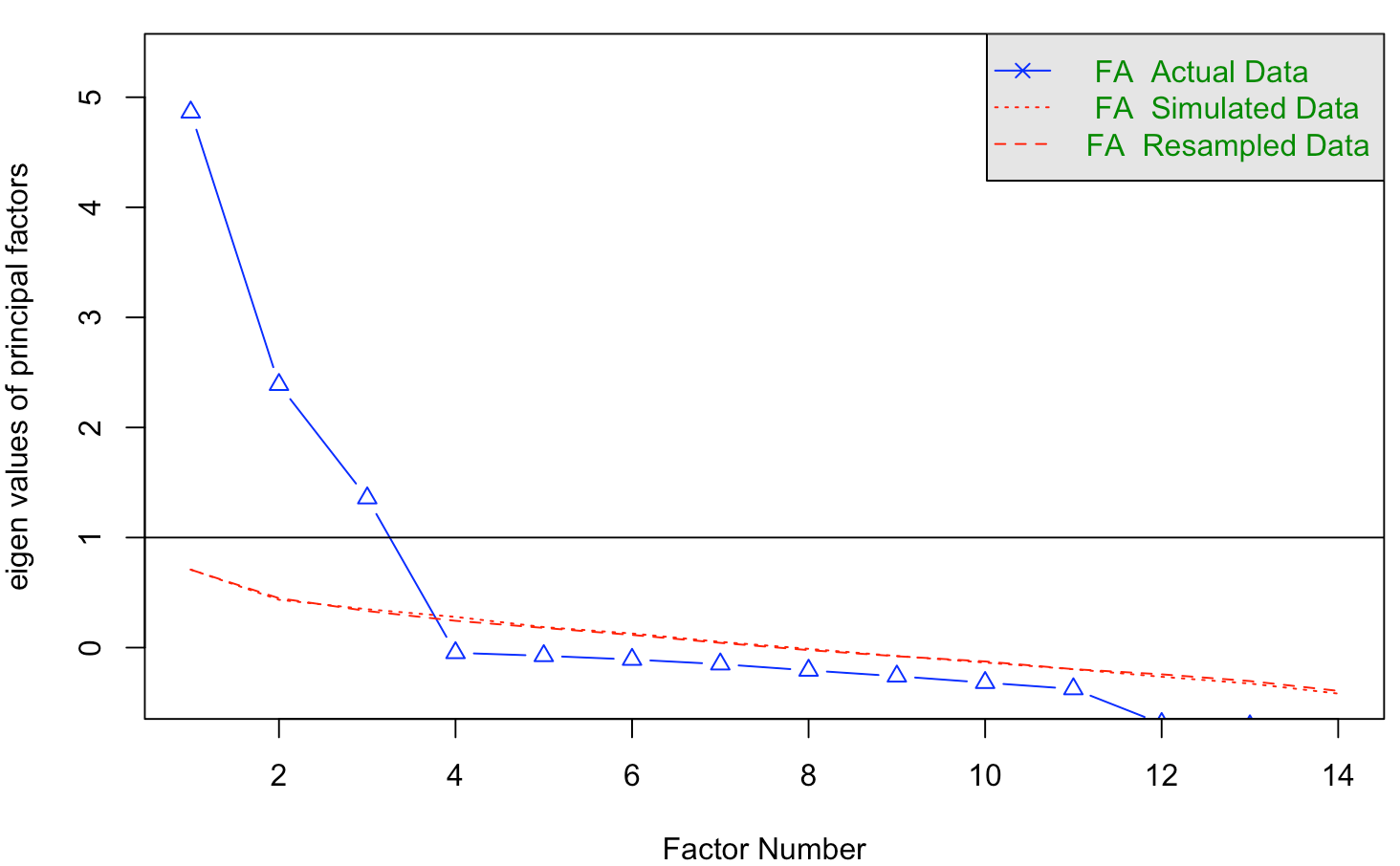


Figure S2. Parallel analysis scree plot for men


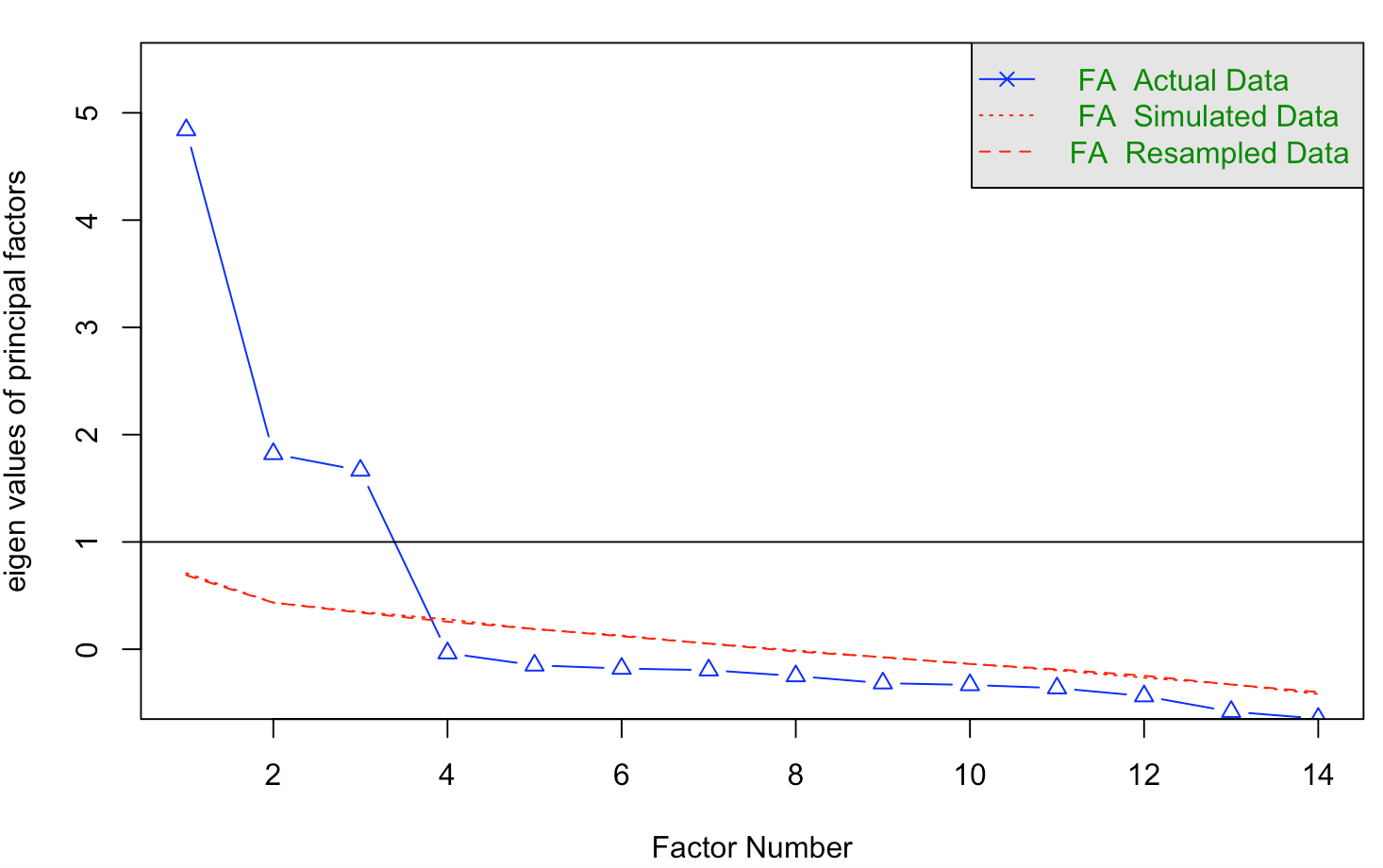


Table S1. Hierarchical linear regressions using the Chinese-Body Talk Scale to describe unique variance in body dissatisfaction and disordered eating in women (*N* = 300)

|  | *F* | Total *R^2^* | $\Delta$ *R^2^* | $\beta$ |
| --- | --- | --- | --- | --- |
| Criterion 1: EDI-BD-Body fat dissatisfaction |  |  |  |  |
| Step 1 | 152.53^***^ | .60^***^ | - |  |
| Age |  |  |  | -.11^**^ |
| BMI |  |  |  | .37^***^ |
| NBTS-Negative body talk |  |  |  | .57^***^ |
| Step 2 | 150.58^***^ | .75^***^ | .15^***^ |  |
| Age |  |  |  | -.05 |
| BMI |  |  |  | .25^***^ |
| NBTS-Negative body talk |  |  |  | .29^***^ |
| C-BTS-Negative fat talk |  |  |  | .14^**^ |
| C-BTS-Negative muscle talk |  |  |  | -.01 |
| C-BTS-Positive body talk |  |  |  | -.44^***^ |
| Criterion 2: FMS-Drive for muscularity |  |  |  |  |
| Step 1 | 14.93^***^ | .12^***^ | - |  |
| Age |  |  |  | .10 |
| BMI |  |  |  | -.13^*^ |
| NBTS-Negative body talk |  |  |  | .38^***^ |
| Step 2 | 56.36^***^ | .53^***^ | .40^***^ |  |
| Age |  |  |  | .05 |
| BMI |  |  |  | -.11^*^ |
| NBTS-Negative body talk |  |  |  | .33^***^ |
| C-BTS-Negative fat talk |  |  |  | .05 |
| C-BTS-Negative muscle talk |  |  |  | .63^***^ |
| C-BTS-Positive body talk |  |  |  | .09 |
| Criterion 3: EDE-QS-Thinness-oriented disordered eating |  |  |  |  |
| Step 1 | 88.53^***^ | .47^***^ | - |  |
| Age |  |  |  | -.07 |
| BMI |  |  |  | .11^*^ |
| NBTS-Negative body talk |  |  |  | .64^***^ |
| Step 2 | 48.79^***^ | .50^***^ | .03^**^ |  |
| Age |  |  |  | -.06 |
| BMI |  |  |  | .04 |
| NBTS-Negative body talk |  |  |  | .49^***^ |
| C-BTS-Negative fat talk |  |  |  | .25^**^ |
| C-BTS-Negative muscle talk |  |  |  | -.04 |
| C-BTS-Positive body talk |  |  |  | .004 |
| Criterion 4: MOET-muscularity-oriented disordered eating |  |  |  |  |
| Step 1 | 54.43^***^ | .35^***^ | - |  |
| Age |  |  |  | .05 |
| BMI |  |  |  | -.09 |
| NBTS-Negative body talk |  |  |  | .62^***^ |
| Step 2 | 39.50^***^ | .44^***^ | .09^***^ |  |
| Age |  |  |  | .03 |
| BMI |  |  |  | -.12^*^ |
| NBTS-Negative body talk |  |  |  | .51^***^ |
| C-BTS-Negative fat talk |  |  |  | .26^**^ |
| C-BTS-Negative muscle talk |  |  |  | .21^***^ |
| C-BTS-Positive body talk |  |  |  | .16^**^ |
| Criterion 5: BAS-2-Body appreciation |  |  |  |  |
| Step 1 | 55.37^***^ | .35^***^ | - |  |
| Age |  |  |  | .13^**^ |
| BMI |  |  |  | -.19^***^ |
| NBTS-Negative body talk |  |  |  | -.50^***^ |
| Step 2 | 81.22^***^ | .62^***^ | .27^***^ |  |
| Age |  |  |  | .07 |
| BMI |  |  |  | -.09^*^ |
| NBTS-Negative body talk |  |  |  | -.25^***^ |
| C-BTS-Negative fat talk |  |  |  | .05 |
| C-BTS-Negative muscle talk |  |  |  | -.05 |
| C-BTS-Positive body talk |  |  |  | .62^***^ |
| Criterion 6: FAS-Functionality appreciation |  |  |  |  |
| Step 1 | 7.26^***^ | .06^***^ | - |  |
| Age |  |  |  | .09 |
| BMI |  |  |  | -.10 |
| NBTS-Negative body talk |  |  |  | -.20^**^ |
| Step 2 | 7.91^***^ | .12^***^ | .07^***^ |  |
| Age |  |  |  | .05 |
| BMI |  |  |  | -.05 |
| NBTS-Negative body talk |  |  |  | -.11 |
| C-BTS-Negative fat talk |  |  |  | .05 |
| C-BTS-Negative muscle talk |  |  |  | .05 |
| C-BTS-Positive body talk |  |  |  | .31^***^ |
| Criterion 7:BI-AAQ-Body image flexibility |  |  |  |  |
| Step 1 | 86.66^***^ | .47^***^ | - |  |
| Age |  |  |  | .13^**^ |
| BMI |  |  |  | -.17^**^ |
| NBTS-Negative body talk |  |  |  | -.60^***^ |
| Step 2 | 45.32^***^ | .48^***^ | .01 |  |
| Age |  |  |  | .12^**^ |
| BMI |  |  |  | -.12^*^ |
| NBTS-Negative body talk |  |  |  | -.50^***^ |
| C-BTS-Negative fat talk |  |  |  | -.08 |
| C-BTS-Negative muscle talk |  |  |  | .02 |
| C-BTS-Positive body talk |  |  |  | .12^*^ |

Notes: C-BTS = Chinese-Body Talk Scale, NBTS = Negative Body Talk Scale, EDI-BD = Body Dissatisfaction subscale of the Eating Disorder Inventory, FMS = Female Muscularity Scale, EDE-QS = Short Form of the Eating Disorder Examination-Questionnaire, MOET = Muscularity-Oriented Eating Test, BAS-2 = Body Appreciation Scale-2, BI-AAQ = Body Image Acceptance and Action Questionnaire. ^*^ *p* < .05, ^**^ *p* < .01, ^***^ *p* < .001.

Table S2. Hierarchical linear regressions using the Chinese-Body Talk Scale to describe unique variance in body dissatisfaction and disordered eating in men (*N* = 300)

|  | *F* | Total *R^2^* | $\Delta$ *R^2^* | $\beta$ |
| --- | --- | --- | --- | --- |
| Criterion 1: EDI-BD-Body fat dissatisfaction |  |  |  |  |
| Step 1 | 89.30^***^ | .54^***^ | - |  |
| Age |  |  |  | -.15^***^ |
| BMI |  |  |  | .10 |
| MBTS-Fat talk |  |  |  | .70^***^ |
| MBTS-Muscle talk |  |  |  | -.12^**^ |
| Step 2 | 126.26^***^ | .75^***^ | .20^***^ |  |
| Age |  |  |  | -.05 |
| BMI |  |  |  | .24^***^ |
| MBTS-Fat talk |  |  |  |  |
| MBTS-Muscle talk |  |  |  | .31^***^ |
| C-BTS-Negative fat talk |  |  |  | .12^*^ |
| C-BTS-Negative muscle talk |  |  |  | -.004 |
| C-BTS-Positive body talk |  |  |  | -.43^***^ |
| Criterion 2: DMS-Drive for muscularity |  |  |  |  |
| Step 1 | 130.19^***^ | .63^***^ | - |  |
| Age |  |  |  | -.02 |
| BMI |  |  |  | -.06 |
| MBTS-Fat talk |  |  |  | .16^***^ |
| MBTS-Muscle talk |  |  |  | .74^***^ |
| Step 2 | 105.51^***^ | .71^***^ | .08^***^ |  |
| Age |  |  |  | .01 |
| BMI |  |  |  | -.04 |
| MBTS-Fat talk |  |  |  | .11 |
| MBTS-Muscle talk |  |  |  | .44^***^ |
| C-BTS-Negative fat talk |  |  |  | -.03 |
| C-BTS-Negative muscle talk |  |  |  | .42^***^ |
| C-BTS-Positive body talk |  |  |  | -.09^*^ |
| Criterion 3: EDE-QS-Thinness-oriented disordered eating |  |  |  |  |
| Step 1 | 98.07^***^ | .57^***^ | - |  |
| Age |  |  |  | -.07 |
| BMI |  |  |  | .12^*^ |
| MBTS-Fat talk |  |  |  | .65^***^ |
| MBTS-Muscle talk |  |  |  | .14^**^ |
| Step 2 | 71.47^***^ | .62^***^ | .06^***^ |  |
| Age |  |  |  | -.06 |
| BMI |  |  |  | .07 |
| MBTS-Fat talk |  |  |  | .30^***^ |
| MBTS-Muscle talk |  |  |  | .16^**^ |
| C-BTS-Negative fat talk |  |  |  | .38^***^ |
| C-BTS-Negative muscle talk |  |  |  | .03 |
| C-BTS-Positive body talk |  |  |  | -.12^**^ |
| Criterion 4: MOET-muscularity-oriented disordered eating |  |  |  |  |
| Step 1 | 94.97^***^ | .56^***^ | - |  |
| Age |  |  |  | .04 |
| BMI |  |  |  | -.01 |
| MBTS-Fat talk |  |  |  | .55^***^ |
| MBTS-Muscle talk |  |  |  | .41^***^ |
| Step 2 | 56.89^***^ | .57^***^ | .01^*^ |  |
| Age |  |  |  | .04 |
| BMI |  |  |  | -.02 |
| MBTS-Fat talk |  |  |  | .42^***^ |
| MBTS-Muscle talk |  |  |  | .33^***^ |
| C-BTS-Negative fat talk |  |  |  | .14 |
| C-BTS-Negative muscle talk |  |  |  | .12 |
| C-BTS-Positive body talk |  |  |  | -.02 |
| Criterion 5: BAS-2-Body appreciation |  |  |  |  |
| Step 1 | 21.42^***^ | .22^***^ | - |  |
| Age |  |  |  | .22^***^ |
| BMI |  |  |  | -.04 |
| MBTS-Fat talk |  |  |  | -.43^***^ |
| MBTS-Muscle talk |  |  |  | .13^*^ |
| Step 2 | 61.39^***^ | .59^***^ | .37^***^ |  |
| Age |  |  |  | .14^**^ |
| BMI |  |  |  | -.02 |
| MBTS-Fat talk |  |  |  | -.02 |
| MBTS-Muscle talk |  |  |  | -.10 |
| C-BTS-Negative fat talk |  |  |  | -.13 |
| C-BTS-Negative muscle talk |  |  |  | -.08 |
| C-BTS-Positive body talk |  |  |  | .65^***^ |
| Criterion 6: FAS-Functionality appreciation |  |  |  |  |
| Step 1 | 2.78^***^ | .02^*^ | - |  |
| Age |  |  |  | .12 |
| BMI |  |  |  | .03 |
| MBTS-Fat talk |  |  |  | -.12 |
| MBTS-Muscle talk |  |  |  | .14^*^ |
| Step 2 | 7.65^***^ | .14^***^ | .12^***^ |  |
| Age |  |  |  | .06 |
| BMI |  |  |  | .03 |
| MBTS-Fat talk |  |  |  | .06 |
| MBTS-Muscle talk |  |  |  | .26^***^ |
| C-BTS-Negative fat talk |  |  |  | -.02 |
| C-BTS-Negative muscle talk |  |  |  | -.21^*^ |
| C-BTS-Positive body talk |  |  |  | .34^***^ |
| Criterion 7:BI-AAQ-Body image flexibility |  |  |  |  |
| Step 1 | 55.26^***^ | .42^***^ | - |  |
| Age |  |  |  | .05 |
| BMI |  |  |  | -.08 |
| MBTS-Fat talk |  |  |  | -.54^***^ |
| MBTS-Muscle talk |  |  |  | -.22^***^ |
| Step 2 | 41.84^***^ | .49^***^ | .07^***^ |  |
| Age |  |  |  | .03 |
| BMI |  |  |  | -.03 |
| MBTS-Fat talk |  |  |  | -.19^*^ |
| MBTS-Muscle talk |  |  |  | -.29^***^ |
| C-BTS-Negative fat talk |  |  |  | -.33^***^ |
| C-BTS-Negative muscle talk |  |  |  | .03 |
| C-BTS-Positive body talk |  |  |  | .20^***^ |

*Notes:* C-BTS = Chinese Body Talk Scale, NBTS = Negative Body Talk Scale, EDI-BD = Body Dissatisfaction subscale of the Eating Disorder Inventory, FMS = Female Muscularity Scale, EDE-QS = Short Form of the Eating Disorder Examination-Questionnaire, MOET = Muscularity-Oriented Eating Test, BAS-2 = Body Appreciation Scale-2, BI-AAQ = Body Image Acceptance and Action Questionnaire. ^*^ *p* < .05, ^**^ *p* < .01, ^***^ *p* < .001

**The Chinese version of the Body Talk Scale**

**《身体谈论量表中文版》**

**指导语：**

下面陈述是一些有关人们对自己身体评价或态度的话。请仔细阅读后圈出**您在与他人交谈时说这些话（或者表达类似意思的话）**的频率。数字越大代表频率越高，请参考：1-从不，2-很少（每隔几个月1次），3-有时（每月1−3次），4-经常（每周1−3次），5-频繁（每天），6-总是（每天数次）。

**请记住，我们不关注您产生类似想法的频率，我们感兴趣的是您在与他人交谈时您说出这样的话（或者表达类似意思的话）的频率。**

|  | 从  不 | 很  少 | 有  时 | 经  常 | 频  繁 | 总  是 |
| --- | --- | --- | --- | --- | --- | --- |
| 1.我需要减肥。 | 1 | 2 | 3 | 4 | 5 | 6 |
| 2.我觉得自己胖。 | 1 | 2 | 3 | 4 | 5 | 6 |
| 3.我的衣服对我现在的体型来说太紧了。 | 1 | 2 | 3 | 4 | 5 | 6 |
| 4.我应该停止吃让我发胖的食物了。 | 1 | 2 | 3 | 4 | 5 | 6 |
| 5.我需要加强运动来减肥。 | 1 | 2 | 3 | 4 | 5 | 6 |
| 6.我要是有更多肌肉就好了。 | 1 | 2 | 3 | 4 | 5 | 6 |
| 7.我要是更加强壮就好了。 | 1 | 2 | 3 | 4 | 5 | 6 |
| 8.我应该吃能够促进肌肉生长的食物。 | 1 | 2 | 3 | 4 | 5 | 6 |
| 9.我需要更频繁地进行举重来锻炼我的肌肉。 | 1 | 2 | 3 | 4 | 5 | 6 |
| 10.我喜欢我现在的样子。 | 1 | 2 | 3 | 4 | 5 | 6 |
| 11.我对自己的身体感觉良好。 | 1 | 2 | 3 | 4 | 5 | 6 |
| 12.我为我的身体所能做到的感到骄傲。 | 1 | 2 | 3 | 4 | 5 | 6 |
| 13.我对自己的饮食习惯感到满意。 | 1 | 2 | 3 | 4 | 5 | 6 |
| 14.我对自己的运动习惯感到满意。 | 1 | 2 | 3 | 4 | 5 | 6 |
